# The preparation and inception of a National Electronic Register for Cardiovascular Rehabilitation (NERCVR) with initial results, as part of a National Electronic Register for Cardiovascular Diseases (NERCVD)

**DOI:** 10.25122/jml-2025-0112

**Published:** 2025-07

**Authors:** Mihaela Mandu, Gelu Onose, Catalina Liliana Andrei, Andreea Elena Lacraru, Ioana Andone, Aura Spinu, Cristina Popescu, Dragos-Alin Trache, Alexandru Ion, Iulian Nastasa, Liliana Florina Andronache, Dumitru Cristinel Badiu, Stefan Sebastian Busnatu

**Affiliations:** 1 Carol Davila University of Medicine and Pharmacy, Bucharest, Romania; 2 Bagdasar-Arseni Clinical Emergency Hospital, Bucharest, Romania

**Keywords:** National Electronic Register, Cardiovascular Rehabilitation Register, Cardiovascular Rehabilitation, REDCap, NERCVD, National Electronic Register for Cardiovascular Diseases, NERCVR, National Electronic Register for Cardiovascular Rehabilitation, REDCap, Research Electronic Data Capture, EBM, Evidence-Based Medicine, EU, European Union, GDPR, General Data Protection Regulation, EMR, Electronic Medical Records, Acute MI, Acute Myocardial Infarction, BMI, Body Mass Index, RPMB, Rehabilitation, Physical Medicine and Balneoclimatology, ECG, Electrocardiography, SOIT, State Office for Inventions and Trademarks, ACEI, Angiotensin-Converting Enzyme Inhibitors, ARB, Angiotensin II Receptor Blockers, SGLT2, Sodium-Glucose Transport Protein 2, NYHA, New York Heart Association, 6MWT, 6-Minute Walk Test, 10MWT, 10-Meter Walk Test, TUG, Timed Up and Go Test, B-ADL, Basic Activities of Daily Living, ESR, Erythrocyte Sedimentation Rate, INR, International Normalized Ratio, aPTT, Activated Partial Thromboplastin Time

## Abstract

A review of the literature and current practice shows that Romania lacks both a National Electronic Register for Cardiovascular Diseases (NERCVD) and a dedicated National Electronic Register for Cardiovascular Rehabilitation (NERCVR). Such registers are increasingly essential in the digital era, particularly within the European Union, where several member states (e.g., Sweden, Germany, the Netherlands) already operate national cardiovascular registries. To identify a feasible IT architecture for a Romanian registry, we assessed available platforms and selected the Research Electronic Data Capture (REDCap) web platform as the most suitable, based on its functionality, infrastructure requirements, and flexibility. Using REDCap, we designed the project “Cardiovascular Rehabilitation and Secondary Prevention” and configured a proof-of-concept NERCVR. We managed to configure, taking into account previous experiences in the field presented in the discussions, a demonstrative model - proof of concept type - of the National Electronic Register for Cardiovascular Rehabilitation, with other types of data categories and related digital components to be created in the future, thus concluding the final form of a National Electronic Register for Cardiovascular Diseases.

## INTRODUCTION

Medicine today is both evidence-based (EBM) and personalized, tailored to each patient’s needs, aligning with the ultramodern paradigm of “P4 Medicine: Preventive, Predictive, Participatory, and Personalized” [1,2]. The concept of EBM emerged at the end of the 19^th^ century and, like many new ideas, was not immediately embraced. Through the sustained efforts of pioneers such as Professor Archibald Cochrane, considered the 'father' of EBM [3], and Canadian physician Dr. Gordon Guyatt [4], the concept gradually gained wide acceptance and is now applied across much of the world [5].

Beginning in the 1950s, electronic medical databases were developed, which today can be accessed—under specific conditions—by physicians and other biomedical professionals worldwide [6]. The problem of collecting and storing, especially large amounts of information, *sine qua non* necessary for valid and contributory research, is not simple. It raises multifaceted issues, ranging from effective accessibility and the technical and human capabilities required, to bioethical considerations (see also the General Data Protection Regulation, GDPR [7]) and the indispensable supporting infrastructure.

Regarding the electronic registers that are increasingly discussed in the literature, these are defined as follows: “*a database of identifiable persons containing a clearly defined set of health and demographic data collected for a specific public health purpose*” [8]. Initially, such registers were used to provide information on epidemiological aspects and to better control the evolution of diseases. Over time, however, their scope has expanded, and they are now used for broader purposes, including the implementation of healthcare prevention programs [8].

The existence of such registers is undoubtedly essential, particularly in the era of digitalization of medical services and in the context of Romania’s membership in the European Union (EU), where several member states, such as Sweden, Germany, and the Netherlands, already operate similar registries [9].

Another characteristic in the literature mentions that medical records contain information regarding: “patient care, public health, technology assessment and research” [8]. These electronic medical registers must include patients with specific characteristics that meet the registry’s criteria, focus on a clearly defined population, and rely on a complex infrastructure to ensure that the information is continuously updated [8]. The main challenge with such registers lies in data collection. In order for such a register to exist, an entire team is needed to fill in and update information in real time. Moreover, the General Data Protection Regulation (GDPR) must be strictly observed [10]. Given the rising number of cyberattacks, robust IT security measures are essential to protect sensitive personal health data. Naturally, these requirements also result in significant costs [11].

With the promotion and expansion of digitalized health systems, the implementation of electronic medical records (EMRs) for patients has become an important objective. The use of EMRs could greatly facilitate data collection for electronic registers. Several studies support the efficiency of digital patient records; however, from a legal standpoint, such systems are not yet fully accepted in Romania, as many aspects of this technology, particularly related to data security and legislative adaptation, require further refinement [12].

Moreover, in the digital era we live in, especially after the COVID-19 pandemic, there is great emphasis on telemedicine, and practically, EMRs could, to a large extent, be completed without direct patient contact [13]. At the same time, by using EMRs, physical contact between the doctor and the patient would be greatly limited. However, no matter how much indirect communication with patients is facilitated by technical means, direct doctor–patient contact remains highly significant, with the objective physical examination being indispensable in the diagnostic and treatment process. This relationship also creates a sense of “empathy, warmth, and connection that cannot be replicated through digital means” [14]. There are, of course, clear benefits to telemedicine: it facilitates access to medical services for people in rural areas, leading to a reduction in healthcare costs, and it also saves time for both patients and medical personnel, time that has become an increasingly valuable resource today [15].

An increasing number of countries in the EU and worldwide have electronic registers for various categories of diseases. In Romania, such national electronic registers have also been introduced, for example, the Romanian Register for Rheumatic Diseases and the Romanian Cancer Register [16-18].

However, our review of the literature shows that Romania still lacks a National Electronic Register for Cardiovascular Diseases and, consequently, for patients undergoing cardiovascular rehabilitation. Furthermore, there is no national standardized program for cardiovascular rehabilitation. In this context, we reiterate and emphasize the contribution of the academic clinics of Neuromuscular Rehabilitation and Cardiology at Bagdasar Arseni Clinical Emergency Hospital, which have developed a structured and standardized cardiovascular rehabilitation protocol that we aim to promote at the national level [9].

## MATERIAL AND METHODS

For the preparation of the National Electronic Register for Cardiovascular Rehabilitation (NERCVR), we encountered multiple and significant challenges. From a technical perspective, the primary requirement was a secure, user-friendly IT system for physicians who would continuously collect specific medical data, one that also minimizes costs. In addition to financial considerations, the project required (and continues to require) a multidisciplinary team consisting of primary care physicians, rehabilitation and cardiology specialists, resident physicians in these fields, IT specialists, and, importantly, the support of the authorities for the implementation and potential expansion of this register at the national level [11].

In this context, with the support of the IT department of Carol Davila University of Medicine and Pharmacy, Bucharest (UMFCD), we initiated the development of this register using the Research Electronic Data Capture (REDCap) web platform, which is already in use within our university. This web platform was designed in the United States of America (USA), and it is widely used in academic settings internationally, precisely because it meets the technical quality aspects necessary for collecting medical data and beyond [19]. The support of UMFCD was crucial for this project, as access to the platform is restricted to users affiliated with institutions that are part of the REDCap consortium. Moreover, the system requires an administrator to provide user support and maintain system updates [19]. REDCap is web-based, requiring no software installation, only an electronic device with internet access [19]. To further facilitate its use, several universities and institutions worldwide have published guides to help users access and operate the REDCap platform [20-22].

**Figure 1:**
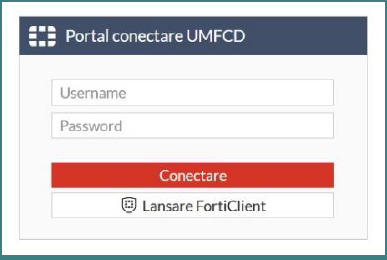
UMFCD login portal for accessing the REDCap platform using institutional credentials

The first step in using this IT platform is to access the University’s login portal and enter the credentials provided by the institution (Figure 1).

**Figure 2:**
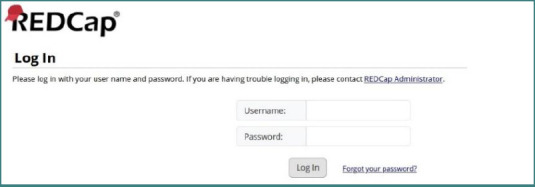
REDCap web interface for entering user credentials to access the platform

Afterward, each user logs in with their credentials to access the REDCap platform (Figure 2).

When selecting REDCap for developing the National Electronic Register for Cardiovascular Rehabilitation, we also considered the need for language adaptability. As this is a Romanian national electronic register, it must support and display data in Romanian, ensuring proper usability for all stakeholders.

## RESULTS

**Figure 3:**
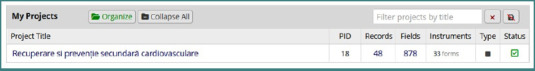
Project “Cardiovascular Rehabilitation and Secondary Prevention” created in REDCap

On the REDCap platform, we created the project entitled “Cardiovascular Rehabilitation and Secondary Prevention” (Figure 3). Before configuring any project, REDCap requires users to define its purpose, with the following options available: *Practice/Just for fun, Operational Support, Research, Quality Improvement*, and *Others* [21]. For our project, we selected “Research” as the primary purpose.

Data collections can be customized according to the needs of the project, which is a great convenience for users in terms of having a unified database adapted to the requirements of a good project, while also having good control over the database. All data collections included can be renamed, deleted, copied, reordered, and thus can be updated as needed.

Since cardiovascular pathology is extensive, as a first step toward developing the national register—and to remain within the reasonable scope of this article—we included only data fields capturing specific information about patients who have had an acute myocardial infarction (acute MI). The project will later be expanded to include other major cardiovascular conditions.

For the development of the NERCVR, we configured the following data collections: Personal/individual data, Infarction - initial cardiologic assessment, Infarction - initial echocardiographic assessment, Infarction - initial angiographic assessment, Infarction - initial non-invasive angiographic assessment, Infarction - initial stress test, Infarction - initial nutritional assessment, Infarction - initial rehabilitation assessment, Infarction - database throughout hospitalization until recovery, Infarction - rehabilitation assessment until discharge, Infarction - stress test reassessment 1, Infarction - cardiologic reassessment 1, Infarction - echocardiographic reassessment 1, Infarction - stress test reassessment 2, Infarction - cardiologic reassessment 2, Infarction - echocardiographic reassessment 2, Infarction - nutritional reassessment, Infarction - final cardiologic assessment, Infarction - additional information.

Each of these data collections contains a set of landmark variables, which can be configured and adapted according to project requirements [20]. The types of data collections that REDCap provides are presented in Table 1.

**Table 1:** Field types available in REDCap for completing data collections

Text Box (Short Text, Number, Date/Time, E-mail, Phone number, etc.)
Notes Box (Paragraph Text)
Calculated Field
Multiple Choice – Radio Buttons (Single Answer)
Checkboxes (Multiple Answer)
Yes-No
True-False
Signature (draw signature with mouse or finger)
File Upload (for users to upload files)
Slider / Visual Analog Scale
Descriptive Text
Begin New Section (with optional text

As previously mentioned, the REDCap platform we have chosen and designated for use in our project provides sufficient facilities and options for collecting and sorting biomedical data, which can be used effectively for the development and implementation of NERCVR.

Moreover, without any need to be modest, we emphasize the substantial effort invested in ordering, reorganizing, and systematizing data structures to provide a coherent and well-structured medical database architecture. This meticulous approach aimed at surfacing and appropriately positioning the options in relation to the diverse and sometimes syncretic range of options existing on the platform.

At the time of data acquisition, REDCap allows the addition of related information in multiple formats (Date, Time, Email, Phone Number, etc.). It also includes a “Signature” field, enabling patients to electronically sign their consent for enrollment (Table 1). This ensures that the database can incorporate personal and legal information in a unified and precise configuration.

For the “Personal/Individual Data” collection, we used a combination of text boxes, radio buttons, checkboxes, sliders, calculated fields, and file uploads, as detailed in Table 2. REDCap also provides the option to export all data in PDF format for documentation and review purposes.

**Table 2:** Personal/individual data fields used in the NERCVR project

Field Label	Field Type	Choices	Required?	Comments
ID	Automatic number		Yes	Automatically assigns each patient an identification number
Last Name	Text Box - Short Text		Yes	
First Name	Text Box - Short Text		Yes	
Consent Information	File Upload		Yes	Scanned patient consent is added
Signature	Signature (draw signature with mouse or finger)		No	The patient's electronic signature can be added
Study entry date	Text Box - Date		Yes	Enrollment date validation (D-M-Y)
Phone number	Text Box – Phone number		Yes	Identification date
Place of living	Multiple Choice – Radio Buttons (Single Answer)	1 = Countryside 2 = Urban	Yes	
Date of birth	Text Box - Date		Yes	Validation of date of birth (D-M-Y)
Age (years)	Calculated Field		Yes	Automatically calculates age based on date of birth
Level of education	Multiple Choice – Radio Buttons (Single Answer)	1 = No education 2 = Primary school 3 = Middle school 4 = High school 5 = Faculty	Yes	
Working status	Multiple Choice – Radio Buttons (Single Answer)	1 = Employee 2 = Unemployed 3 = Pensioner	No	
Ethnicity	Multiple Choice – Radio Buttons (Single Answer)	1 = Romanian 2 = Hungarian 3 = German 4 = Romani 5 = Others	No	
Gender	Multiple Choice – Radio Buttons (Single Answer)	1 = Female 2 = Male 3 = Other	Yes	
Marital status	Multiple Choice – Radio Buttons (Single Answer)	1 = Single 2 = In a relationship 3 = Married 4 =Divorced 5 =Widower	No	
Number of births	Text Box – Number		No	Minimum 0 Maximum 10
Number of pregnancies	Text Box – Number		No	Minimum 0 Maximum 20
Family history of vascular disease	Check boxes (Multiple Answer)	1 = No 2 = Myocardial infarction 3 = Stroke 4 = Sudden death 5 = Others	Yes	
Personal pathological history	Check boxes (Multiple Answer)	1 = High blood pressure 2 = Dyslipidemia 3 = Obesity 4 = Angina pectoris 5 = Myocardial infarction 6 = Diabetes mellitus 7 = Atherosclerosis obliterans 8 = Others 9 = No	Yes	
Patient's mood at cardiology admission	Slider / Visual Analogue Scale	0 = Depressed 50 = Indifferent 100 = Motivated	Yes	

To calculate age from the date of birth, we used dedicated calculation equations. In the Personal Pathological History section, we implemented multiple-choice fields with several possible answers, focusing primarily on cardiovascular risk factors: *The options* included: *hypertension (high blood pressure), dyslipidemia, obesity, angina pectoris, diabetes mellitus, sedentary lifestyle, and others*.

Additionally, we created a data collection focused on the initial nutritional assessment, using specific equations to calculate parameters such as body mass index (BMI), body fat percentage, and muscle mass.

Since cardiovascular pathology is extensive and Romania still lacks a National Electronic Register for Cardiovascular Diseases (NERCVD), we faced the challenge of creating a database tailored to the subcategories of this pathology. Given that establishing such a register is a particularly complex process, requiring substantial time and multi-level resources, and considering the wide spectrum of cardiovascular diseases (including patients eligible for our Cardiovascular Rehabilitation program), we decided to begin sectorally. Thus, as a first step, we focused on a relevant subgroup—patients with acute myocardial infarction—to later expand the register to other categories of cardiovascular patients within the future NERCVD framework. Accordingly, we created the data collection entitled “Infarction – Initial Cardiological Evaluation” (Table 3), which includes key information about the patient’s status at the time of the infarction, such as the date of the acute event, symptoms upon presentation to the Cardiology Emergency Room, home medications, blood test results, and investigations (e.g., ECG, electrical conversion, etc.).

**Table 3:** Data collection “Infarction – initial cardiologic assessment”

Field Label	Field Type	Choices	Required?	Comments
Date of occurrence of the acute event	Text Box - Date		Yes	Validation – Date (D-M-Y)
Clinical symptoms when admitted to the cardiology ward	Checkboxes (Multiple Answer)	1 = Dyspnea 2 = Angina pectoris 3 = Palpitations 4 = Syncope 5 = Fatigue 6 = Other	Da Da Yes	
NYHA heart failure degree – upon admission	Multiple Choice – Radio Buttons (Single Answer)	1 = I 2 = II 3 = III 4 = IV	Yes	
NYHA heart failure grade – at discharge	Multiple Choice – Radio Buttons (Single Answer)	1 = I 2 = II 3 = III 4 = IV	Yes	
Duration of pain (hours)	Text Box - Number		Yes	Minimum 0 Maximum 72
Duration of symptoms - other than chest pain (hours)	Text Box - Number		Yes	Minimum 0 Maximum 72
Treatment at home before reaching cardiology	Check boxes (Multiple Answer)	1 = Beta-blockers 2 = ACEI/ARB 3 = Calcium channel blockers 4 = Diuretics 5 = Antiaggregant 6 = Anticoagulants 7 = Angina Relievers 8 = Statins 9 = Fibrates 10 = No treatment	Yes	
Active smoker	Multiple Choice – Radio Buttons (Single Answer)	1 = Yes 2 = No	Yes	
Pack/ Year (Cigarettes)	Text Box - Number		No	Minimum 0 Maximum 150
Before Myocardial infarction: LDL (mg/dl)	Text Box - Number		No	Minimum 0 Maximum 999
Before myocardial infarction: HDL (mg/dl)	Text Box - Number		No	Minimum 0 Maximum 999
Before Myocardial infarction: Total Cholesterol (mg/dl)	Text Box - Number		No	Minimum 0 Maximum 999
Before Myocardial infarction: Triglycerides (mg/dl)	Text Box - Number		No	Minimum 0 Maximum 999
Apo-B (mg/dl)	Text Box - Number		No	
Lipoprotein A (mg/dl)	Text Box - Number		No	
Before Myocardial infarction: Na (mmol/L)	Text Box - Number		No	
Before Myocardial infarction: K (mmol/L)	Text Box - Number		No	
Before Myocardial infarction: Cl (mmol/L)	Text Box - Number		No	
Hemoglobin value upon admission (g/dl)	Text Box - Number		Yes	Minimum 1 Maximum 20
First Day Hemoglobin (g/dl)	Text Box - Number		No	Minimum 1 Maximum 20
Thrombolysis	Multiple Choice – Radio Buttons (Single Answer)	1 = Yes 2 = No	No	
Thrombolysis criteria	Multiple Choice – Radio Buttons (Single Answer)	1 = Positive 2 = Negative	No	
Killip	Multiple Choice – Radio Buttons (Single Answer)	1 = 1 2 = 2 3 = 3 4 = 4	No	
Loading dose of Plavix/Brilique	Multiple Choice – Radio Buttons (Single Answer)	1 = Plavix 2 = Brilique	No	
ECG	Check boxes (Multiple Answer)	1 = Overelevation ST 2 = Under-elevation ST 3 = Q wave 4 = Pathological T wave 5 = Arrythmia 6 = Others	No	
Pre-infarction complications– Supraventricular arrhythmias	Check boxes (Multiple Answer)	1 = Supraventricular paroxysmal tachycardia 2 = Atrial fibrillation 3 = Atrial Flutter 4 = Supraventricular extra systole	No	
Acute MI complications- ventricular arrhythmia	Check boxes (Multiple Answer)	1 = Unsustained ventricular tachycardia 2 = Sustained ventricular tachycardia 3 = Ventricular extrasystole 4 = Ventricular fibrillation 5 = Apex torsade 6 = Ventricular flutter 7 = Cardiac arrest	No	
Electrical conversion	Multiple Choice – Radio Buttons (Single Answer)	1 = Yes 2 = No	No	
Cardiac arrest	Multiple Choice – Radio Buttons (Single Answer)	1 = Yes 2 = No	No	
Symptoms throughout the cardiology admission	Check boxes (Multiple Answer)	1 = Angina pectoris 2 = Syncope 3 = Palpitations 4 = Bleeding	No	
Bleeding?	Multiple Choice – Radio Buttons (Single Answer)	1 = Yes 2 = No	No	
Treatment recommended upon discharge	Check boxes (Multiple Answer)	1 = Beta blockers 2 = Antiaggregant 3 = Double anti-aggregation 4 = Anticoagulant 5 = Statins 6 = ACEI or ARB 7 = Calcium channel blockers 8 = Central alpha blockers 9 = SGLT2 Inhibitor 10 = Loop diuretics 11 = Potassium-saving diuretics 12 =Thiazide diuretics 13 = Nitrates 14 = Beta-oxidase inhibitors 15 = Others	No	

Subsequently, after being assessed, treated, and stabilized, medically fit patients are enrolled in the Cardiovascular Rehabilitation Program initiated at “Bagdasar Arseni” Clinical Emergency Hospital. Following an initial cardiopulmonary exercise test, patients undergo a comprehensive assessment by the Rehabilitation, Physical Medicine, and Balneoclimatology (RPMB) team. As part of this evaluation, physicians use standardized assessment grids to precisely and uniformly determine patients’ exercise tolerance. These include: 30 Second Sit to Stand Test, which evaluates exercise endurance and also helps assess fall risk [23], 6-Minute Walk Test [24], and 10-Meter Walk Test [25]. We also included scales to quantify the degree to which these cardiovascular conditions impact the quality of life (QoL) of patients. The initial RPMB evaluation also includes specific blood tests, as well as an electrocardiographic evaluation (Table 4). To monitor progress, all these measurements are repeated at the completion of the cardiovascular rehabilitation program.

**Table 4:** Data collection “Infarction – initial RPMB assessment”

Field Label	Field Type	Choices	Required?	Comments
Sit to Stand Test - Number of lifts	Text Box – Number		Yes	Minimum 0 Maximum 30
Sit to Stand Test - Degree of resistance to effort and risk of falling	Calculated Field	1 = Normal resistance – low risk of falling 2 = Low resistance – increased risk of falling	Da DaN Yes Yes	
6MWT - Distance	Text Box – Number		Yes	Minimum 0 Maximum 1000
6MWT symptomatic?	Multiple Choice – Radio Buttons (Single Answer)	1 = Yes 2 = No	Yes	
Modified Borg Scale	Slider / Visual Analogue Scale	0 = Rest 5 = Hard 10 = Maximum effort	Yes	
10 MWT – medium speed comfort (m/s)	Text Box – Number		Yes	Minimum 0.1 Maximum 3.0
10 MWT – medium speed rapid (m/s)	Text Box – Number		Yes	Minimum 0.1 Maximum 4.0
TUG - Risk of falling	Multiple Choice – Radio Buttons (Single Answer)	1 = < 12 seconds 2 = >= 12 seconds	Yes	
Katz Index (B-ADL)	Multiple Choice – Radio Buttons (Single Answer)	1 = 6 points: independent 2 = 4 points: moderately dependent 3 = 2 points: high dependence grade 4 = 0 points: total dependency	Yes	
Minnesota	Multiple Choice – Radio Buttons (Single Answer)	1 = 0 points: No impact on QoL 2 = 1-24 points: Mild impact on QoL 3 = 25-49 points: Moderate impact on QoL 4 = 50-74 points: Severe impact on QoL 5 = 75-105 points = Major impact on QoL	Yes	
SF-36 - Quality of life	Multiple Choice – Radio Buttons (Single Answer)	1 = 3000-3600 Excellent 2 = 2450-2999 Good 3 = 1750-2449 Moderate 4 = < 1750 Severely Affected	Yes	
Serum albumin (g/dL)	Text Box – Number		No	Minimum 3 Maximum 5
Total proteins (g/dl)	Text Box – Number		No	
Total Cholesterol (mg/dL)	Text Box – Number		No	Minimum 100 Maximum 600
LDL cholesterol (mg/dL)	Text Box – Number		No	Minimum 1 Maximum 300
HDL cholesterol (mg/dL)	Text Box – Number		No	Minimum 1 Maximum 300
Triglycerides	Text Box – Number		No	Minimum 1 Maximum 500
GOT/GPT	Text Box – Number		No	Minimum 1 Maximum 500
Fasting glycemia	Text Box – Number		No	Minimum 1 Maximum 500
CK (u/l)	Text Box – Number		No	Minimum 1 Maximum 1000
CK MB (u/l)	Text Box – Number		No	Minimum 1 Maximum 9999
Urea (mg/dL)	Text Box – Number		No	Minimum 0.5 Maximum 20
Creatinine (mg/dL)	Text Box – Number		No	Minimum 0.5 Maximum 20
Uric acid (mg/dl)	Text Box – Number		No	Minimum 1 Maximum 120
Na (mmol/l)	Text Box – Number		No	Minimum 1 Maximum 200
K (mmol/l)	Text Box – Number		No	Minimum 1 Maximum 10
Cl (mmol/L)	Text Box – Number		No	Minimum 1 Maximum 200
Ca (mg/dl)	Text Box – Number		No	Minimum 1 Maximum 20
Iron (microgram/dl)	Text Box – Number		No	Minimum 1 Maximum 999
Magnesium (mg/dl)	Text Box – Number		No	Minimum 0 Maximum 100
Fibrinogen (mg/dl)	Text Box – Number		No	Minimum 1 Maximum 999
ESR	Text Box – Number		No	Minimum 1 Maximum 999
Hemoglobin (g/dl)	Text Box – Number		No	Minimum 1 Maximum 20
Number of leukocytes	Text Box – Number		No	
Number of platelets	Text Box – Number		No	
INR	Text Box – Number		No	Minimum 1 Maximum 10
aPTT (s)	Text Box – Number		No	Minimum 10 Maximum 100
ECG - Rhythm	Multiple Choice – Radio Buttons (Single Answer)	1 = Sinus rhythm 2 = Atrial fibrillation 3 = Atrial Flutter atrial 4 = Junction rhythm 5 = Pacing	Yes	
ECG - Heart rate	Multiple Choice – Radio Buttons (Single Answer)	1 = 50-69 2 = 70-99 3 = >=100	Yes	
ECG - ST-T changes	Multiple Choice – Radio Buttons (Single Answer)	1 = Yes 2 = No	Yes	
ECG - wave Q	Multiple Choice – Radio Buttons (Single Answer)	1 = Yes 2 = No	Yes	
ECG – other changes			No	

During the rehabilitation program, the information collected by the sensor ergometric bicycle is added to the NERCVR for each patient enrolled in the program (Table 5). Because the duration of the program is different from case to case, the data from the first seven days of rehabilitation and the last day (regardless of which day it is) will be collected in the register. Therefore, only patients who have completed at least eight days of training on the sensor ergometric bicycle will be included in the register.

**Table 5:** Data collection “Infarction – database throughout hospitalization until rehabilitation”

Field Label	Field Type	Choices	Required?	Comments
Date of the first day of training	Text Box – Number		Yes	
Number of days since acute MI until the beginning of the rehabilitation programme	Calculated Field		Yes	
The number of days of force training	Text Box – Number		Yes	Minimum 7 Maximum 99
Force training (%1-RM)	Multiple Choice – Radio Buttons (Single Answer)	1 = Yes 2 = No	Yes	
Force training – number of sessions	Slider / Visual Analogue Scale	0 = Rest 5 = Hard 10 = Maximum effort	Yes	
Number of days of training on the ergometric bike	Text Box – Number		Yes	
Warm-up protocol – maximum W	Text Box – Number		No	
Warm-up protocol - time (s)	Text Box – Number		No	
Plateau protocol – maximum W	Text Box – Number		No	
Plateau protocol - time (s)	Text Box – Number		No	
Recovery protocol - maximum W	Text Box – Number		No	
Protocol – total time (s)	Text Box – Number		No	
First day – effort load (W)	Text Box – Number		Yes	
First day – effort load (METs)	Text Box – Number		Yes	
First day – maximum cardiac rhythm (bpm)	Text Box – Number		No	
First day – maximum BP (mmHg)	Text Box – Number		No	
First day – used energy (Kj)	Text Box – Number		No	
First day – Borg Scale (RPE)	Slider / Visual Analogue Scale	6-8 = Very easy 12-13 = Moderate 20 = Maximum	No	
First day - DUKE Score	Slider / Visual Analogue Scale	1 5 10	No	
First day – Total bicycle time	Text Box – Number		Yes	
First day – Was the rehabilitation program discontinued early?	Multiple Choice – Radio Buttons (Single Answer)	1 =Yes 2 = No	No	
First day – symptoms during training	Multiple choice variable	1 = Angina during exercise training 2 = Angina at the end of exercise training 3 = Dyspnea during training 4 = Dyspnea at the end of training 5 = Syncope 6 = Absent	Yes	
The second day – effort load (W)	Text Box – Number		Yes	
The second day - effort load (METs)	Text Box – Number		Yes	
The second day – maximum cardiac rhythm (bpm)	Text Box – Number		No	
The second day – maximum BP (mmHg)	Text Box – Number		No	
The second day – used energy (Kj)	Text Box – Number		No	
The second day - Borg Scale (RPE)	Slider / Visual Analogue Scale	6-8 = Very easy 12-13 = Moderate 20 = Maximum	No	
The second day – DUKE Score	Slider / Visual Analogue Scale	1 5 10	No	
The second day – Total bicycle time	Text Box – Number		Yes	
The second day - Was the rehabilitation program discontinued early?	Multiple Choice – Radio Buttons (Single Answer)	1 = Yes 2 = No	No	
The second day – symptoms during training	Multiple choice variable	1 = Angina during exercise training 2 = Angina at the end of exercise training 3 = Dyspnea during training 4 = Dyspnea at the end of training 5 = Syncope 6 = Absent	Yes	
The third day – effort load (W)	Text Box – Number		Yes	
The third day - effort load (METs)	Text Box – Number		Yes	
The third day – maximum cardiac rhythm (bpm)	Text Box – Number		No	
The third day – maximum BP (mmHg)	Text Box – Number		No	
The third day – used energy (Kj)	Text Box – Number		No	
The third day – Borg Scale (RPE)	Slider / Visual Analogue Scale	6-8 = Very easy 12-13 = Moderate 20 = Maximum	No	
The third day – DUKE Score	Slider / Visual Analogue Scale	1 5 10	No	
The third day – Total bicycle time	Text Box – Number		Yes	
The third day - Was the rehabilitation program discontinued early?	Multiple Choice – Radio Buttons (Single Answer)	1 = Yes 2 = No	No	
The third day - symptoms during training	Multiple choice variable	1 = Angina during exercise training 2 = Angina at the end of exercise training 3 = Dyspnea during training 4 = Dyspnea at the end of training 5 = Syncope 6 =Absent	Yes	
The fourth day – effort load (W)	Text Box – Number		Yes	
The fourth day - effort load (METs)	Text Box – Number		Yes	
The fourth day – maximum cardiac rhythm (bpm)	Text Box – Number		No	
The fourth day – maximum BP (mmHg)	Text Box – Number		No	
The fourth day – used energy (Kj)	Text Box – Number		No	
The fourth day – Borg Scale (RPE)	Slider / Visual Analogue Scale	6-8 = Very east 12-13 = Moderate 20 = Maximum	No	
The fourth day - DUKE Score	Slider / Visual Analogue Scale	1 5 10	No	
The fourth day – Total bicycle time	Text Box – Number		Yes	
The fourth day - Was the rehabilitation program discontinued early?	Multiple Choice – Radio Buttons (Single Answer)	1 = Yes 2 = No	No	
The fourth day - symptoms during training	Multiple choice variable	1 = Angina during exercise training 2 = Angina at the end of exercise training 3 = Dyspnea during training 4 = Dyspnea at the end of training 5 = Syncope 6 = Absent	Yes	
The fifth day – effort load (W)	Text Box – Number		Yes	
The fifth day – effort load (METs)	Text Box – Number		Yes	
The fifth day – maximum cardiac rhythm (bpm)	Text Box – Number		No	
The fifth day – maximum BP (mmHg)	Text Box – Number		No	
The fifth day – used energy (Kj)	Text Box – Number		No	
The fifth day – Borg Scale (RPE)	Slider / Visual Analogue Scale	6-8 = Very easy 12-13 = Moderate 20 = Maximum	No	
The fifth day – DUKE Score	Slider / Visual Analogue Scale	1 5 10	No	
The fifth day – Total bicycle time	Text Box – Number		Yes	
The fifth day - Was the rehabilitation program discontinued early?	Multiple Choice – Radio Buttons (Single Answer)	1 = Yes 2 = No	No	
The fifth day - symptoms during training	Multiple choice variable	1 = Angina during exercise training 2 = Angina at the end of exercise training 3 = Dyspnea during training 4 = Dyspnea at the end of training 5 = Syncope 6 = Absent	Yes	
The sixth day – effort load (W)	Text Box – Number		Yes	
The sixth day – effort load (METs)	Text Box – Number		Yes	
The sixth day – maximum cardiac rhythm (bpm)	Text Box – Number		No	
The sixth day – maximum BP (mmHg)	Text Box – Number		No	
The sixth day – used energy (Kj)	Text Box – Number		No	
The sixth day – Borg Scale (RPE)	Slider / Visual Analogue Scale	6-8 = Very easy 12-13 = Moderate 20 = Maximum	No	
The sixth day – DUKE Score	Slider / Visual Analogue Scale	1 5 10	No	
The sixth day – Total bicycle time	Text Box – Number		Yes	
The sixth day - Was the rehabilitation program discontinued early?	Multiple Choice – Radio Buttons (Single Answer)	1 = Yes 2 = No	No	
The sixth day - symptoms during training	Multiple choice variable	1 = Angina during exercise training 2 = Angina at the end of exercise training 3 = Dyspnea during training 4 = Dyspnea at the end of training 5 = Syncope 6 = Absent	Yes	
The seventh day – effort load (W)	Text Box – Number		Yes	
The seventh day – effort load (METs)	Text Box – Number		Yes	
The seventh day – maximum cardiac rhythm (bpm)	Text Box – Number		No	
The seventh day – maximum BP (mmHg)	Text Box – Number		No	
The seventh day - used energy (Kj)	Text Box – Number		No	
The seventh day - Borg Scale (RPE)	Slider / Visual Analogue Scale	6-8 = Very easy 12-13 = Moderate 20 = Maximum	No	
The seventh day - DUKE Score	Slider / Visual Analogue Scale	1 5 10	No	
The seventh day – Total bicycle time	Text Box – Number		Yes	
The seventh day - Was the rehabilitation program discontinued early?	Multiple Choice – Radio Buttons (Single Answer)	1 = Yes 2 = No	No	
The seventh day - symptoms during training	Multiple choice variable	1 = Angina during exercise training 2 = Angina at the end of exercise training 3 = Dyspnea during training 4 = Dyspnea at the end of training 5 = Syncope 6 = Absent	Yes	
The last day - effort load (W)	Text Box – Number		Yes	
The last day - effort load (METs)	Text Box – Number		Yes	
The last day - maximum cardiac rhythm (bpm)	Text Box – Number		No	
The last day - maximum BP (mmHg)	Text Box – Number		No	
The last day - used energy (Kj)	Text Box – Number		No	
The last day - Borg Scale (RPE)	Slider / Visual Analogue Scale	6-8 = Very easy 12-13 = Moderate 20 = Maximum	No	
The last day – DUKE Score	Slider / Visual Analogue Scale	1 5 10	No	
The last day - Total bicycle time	Text Box – Number		Yes	
The last day - Was the rehabilitation program discontinued early?	Multiple Choice – Radio Buttons (Single Answer)	1 = Yes 2 = No	No	
The last day - symptoms during training	Multiple choice variable	1 = Angina during exercise training 2 = Angina at the end of exercise training 3 = Dyspnea during training 4 = Dyspnea at the end of training 5 = Syncope 6 = Absent	Yes	

**Figure 4:**
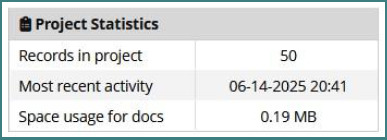
Total number of entries in the NERCVR as of 14.06.2025

Having completed the configuration of NERCVR, we have already introduced 50 patients who have completed the structured Cardiovascular Rehabilitation Program designed and approved as a protocol at Bagdasar-Arseni Clinical Emergency Hospital, Bucharest (Figure 4).

## DISCUSSION

In 2017, our Neuromuscular Rehabilitation Clinic was invited by the Cardiology Clinic, both part of Bagdasar-Arseni Clinical Emergency Hospital, Bucharest, along with several members of the teaching staff at UMFCD, to participate in an international EU-funded project under the Horizon 2020 research program: *Virtual Training Activities for Rehabilitation in the Elderly* (vCare) [26]. This project aimed to develop virtual trainers for personalized rehabilitation programs and home-care methods, representing a first step toward implementing telerehabilitation in Romania [27]. Such a telemedicine system could also play a major role in the secondary prevention of cardiovascular disease [26].

At the same time, we state that our Clinical Department of Neuromuscular Rehabilitation has a very in-depth and already long-standing experience in developing a national electronic register, as it has been involved since 2006 in creating a National Electronic Register for people who have had spinal cord injuries, within the scientific research project entitled: “*The inception of a complex national network, of dynamic clustering of patients with sequelae after spinal cord injuries, dedicated to improving their quality of life, as a contributory approach to the efficiency of specialized medical and social services, in transition*”, project with the Romanian acronym RISCI. This project won the National Competition for Excellence Research (CEEX) launched by the Ministry of Education and Research (MER/ANCS) in 2006 (CEEX contract no. 79/2006, Project Director: Assoc. Prof. Dr. G. Onose) and was later patented in 2011 as a Utility Model by the State Office for Inventions and Trademarks (SOIT).

Our efforts to implement and develop a National Cardiovascular Rehabilitation Program and to set up a National Electronic Register for Cardiovascular Rehabilitation started in 2023 and evolved practically simultaneously with the efforts of the Romanian Government, which recently published the National Strategy for Combating Cardiovascular and Cerebrovascular Diseases 2024-2030. Among its main objectives are: *“the development of an integrated healthcare information system for the prevention and care of cardiovascular and cerebrovascular diseases”* and *“the development of cardiovascular and cerebrovascular rehabilitation in Romania*”.

## CONCLUSION

With the emergence of the COVID-19 pandemic, the concept of digitized health systems and telemedicine has been widely promoted. In such a global context, it is essential to have national electronic registers in various fields of pathology that are useful in collecting, storing and updating medical data to allow access to this information by qualified professionals who wish to conduct clinical trials, as well as to facilitate patients’ access to specialized medical care, including in areas that are physically difficult for patients and/or medical and healthcare personnel.

For the inception of such a register, it was first necessary to establish an IT infrastructure capable of supporting a project of this scale while remaining within a reasonable budget. In addition to technical resources, the development of a national electronic register also requires adequate human and material resources and, importantly, the time, a particularly valuable resource today, needed for its continuous operation. Through the exemplary collaboration between physicians—most of whom are members of the academic staff at UMFCD, Bucharest—from the Clinical Departments of Neuromuscular Rehabilitation and Cardiology at “Bagdasar Arseni” Clinical Emergency Hospital, and with the support of the IT Department of UMFCD, we successfully configured a demonstration model of a National Electronic Register for Cardiovascular Rehabilitation. This model serves as a foundation for incorporating additional data categories and digital components, ultimately leading to the development of a comprehensive National Electronic Register for Cardiovascular Diseases. We hope that the inception and further development of these electronic registers represent a significant step forward for the Romanian healthcare system in addressing cardiovascular diseases, which remain the leading cause of mortality in Romania.

## Personal thanks

The under-development National Electronic Register for Cardiovascular Diseases uses the REDCap portal as its digital infrastructure, based on a mutual agreement with Carol Davila University of Medicine and Pharmacy. This article is part of the overall endeavor towards the achievement of our national electronic register for cardiovascular diseases, which is registered with the State Office for Inventions and Trademarks. Registration Number: f 2025 0105/07.07.2025
